# Anchoring and ordering NGS contig assemblies by population sequencing (POPSEQ)

**DOI:** 10.1111/tpj.12319

**Published:** 2013-10-10

**Authors:** Martin Mascher, Gary J Muehlbauer, Daniel S Rokhsar, Jarrod Chapman, Jeremy Schmutz, Kerrie Barry, María Muñoz-Amatriaín, Timothy J Close, Roger P Wise, Alan H Schulman, Axel Himmelbach, Klaus FX Mayer, Uwe Scholz, Jesse A Poland, Nils Stein, Robbie Waugh

**Affiliations:** 1 Leibniz Institute of Plant Genetics and Crop Plant Research (IPK)D–06466 Seeland OT, Gatersleben, Germany; 2 University of Minnesota, Department of Agronomy and Plant GeneticsSt Paul, MN, 55108, USA; 3 University of Minnesota, Department of Plant BiologySt Paul, MN 55108, USA; 4 Department of Energy Joint Genome Institute2800 Mitchell Drive, Walnut Creek, CA, 94598, USA; 5 Department of Molecular and Cell Biology, University of CaliforniaBerkeley, CA, 94720, USA; 6 HudsonAlpha Institute of BiotechnologyHuntsville, AL, 35806, USA; 7 Department of Botany & Plant Sciences, University of CaliforniaRiverside, CA, 92521, USA; 8 US Department of Agriculture/Agricultural Research Service, Department of Plant Pathology & Microbiology, Iowa State UniversityAmes, IA, 50011–1020, USA; 9 Institute of Biotechnology, University of Helsinki/MTT Agrifood ResearchPO Box 65, 00014, Helsinki, Finland; 10 Munich Information Center for Protein Sequences/Institute of Bioinformatics and Systems Biology, Helmholtz Zentrum MünchenD–85764, Neuherberg, Germany; 11 US Department of Agriculture/Agricultural Research Service, Hard Winter Wheat Genetics Research Unit and Department of Agronomy, Kansas State UniversityManhattan, KS, 65506, USA; 12 Division of Plant Sciences, University of Dundee at the James Hutton InstituteInvergowrie, Dundee, DD2 5DA, UK

**Keywords:** next-generation sequencing, genome assembly, genetic mapping, barley, *Hordeum vulgare*, population sequencing, technical advance

## Abstract

Next-generation whole-genome shotgun assemblies of complex genomes are highly useful, but fail to link nearby sequence contigs with each other or provide a linear order of contigs along individual chromosomes. Here, we introduce a strategy based on sequencing progeny of a segregating population that allows *de novo* production of a genetically anchored linear assembly of the gene space of an organism. We demonstrate the power of the approach by reconstructing the chromosomal organization of the gene space of barley, a large, complex and highly repetitive 5.1 Gb genome. We evaluate the robustness of the new assembly by comparison to a recently released physical and genetic framework of the barley genome, and to various genetically ordered sequence-based genotypic datasets. The method is independent of the need for any prior sequence resources, and will enable rapid and cost-efficient establishment of powerful genomic information for many species.

## Introduction

Next-generation sequencing provides the opportunity to rapidly establish gene space assemblies for virtually any species at relatively low cost. These assemblies consist of tens to hundreds of thousands of short contiguous pieces of DNA sequence (contigs), and often represent only the low-copy portion of the genome. Despite the limitations of such assemblies, they have been widely proposed as surrogates for draft genome sequences for the purposes of gene isolation, genomics-assisted breeding and the assessment of diversity within and between species (Brenchley *et al*., 2012; International Barley Genome Sequencing Consortium, 2012; Guo *et al*., 2013; Xu *et al*., 2013). However, in most cases, particularly those concerning large and complex genomes, they remain disconnected collections of short sequence contigs that are not embedded in a genomic context. Bringing these together into a tentative linear order, or even associating contigs with individual chromosomes or chromosome arms, has been a major and costly undertaking. In a recent example, the International Barley Genome Sequencing Consortium reported the development and use of a BAC-based physical map, BAC end sequences, survey sequences of flow-sorted chromosome arms, fully sequenced BAC clones and conserved synteny to fully contextualize only 410 Mb of genomic sequence from the 5.1 Gb barley genome (International Barley Genome Sequencing Consortium, 2012). These genomic resources provide an established path towards a reference sequence by sequencing a minimum tiling path of overlapping BAC clones hierarchically (Feuillet *et al*., 2012). Development of the necessary resources requires a substantial amount of time, labor and money, which makes this strategy prohibitive for smaller and more poorly resourced research communities, e.g. research in non-model organism or orphan crops. The establishment of a BAC-based reference sequence of the maize genome took approximately 7 years, required the coordinated effort of several laboratories, and cost approximately US $50 million (Chandler and Brendel, 2002; Martienssen *et al*., 2004; Schnable *et al*., 2009). Similarly, the reference sequence of a single 1 Gb chromosome of hexaploid wheat (*Triticum aestivum*) has not been completed 5 years after publication of a physical map (Paux *et al*., 2008).

Emerging technologies such as longer sequence reads (Schadt *et al*., 2010), optical mapping (Lam *et al*., 2012) and novel assembly algorithms (such as ALLPATHS–LG, Gnerre *et al*., 2011)) may speed up the process of data collection and analysis, as well as increasing the contiguity and completeness of whole-genome shotgun (WGS) assemblies, but their applicability to large genomes with abundant sequence repeats (the bane of any assembler), arising from paralogous duplications, repetitive elements, ancestral duplications and polyploidy, remains to be assessed.

It has been common practice to associate mapped genetic markers with sequence resources based on sequence similarity in order to link genetic and physical maps (Chen *et al*., 2002; Wei *et al*., 2007). While the number of BAC contigs on a physical map is in order of thousands, next-generation sequencing (NGS) technology produces hundreds of thousands of sequence contigs. For example, the International Barley Genome Sequencing Consortium (2012) reported an assembly that consists of more than 350 000 contigs longer than 1 kb. The number of markers afforded by conventional genotyping strategies is simply not commensurate with the large number of short sequence contigs.

Several methods for high-throughput genotyping of genetic mapping populations using next-generation sequencing technology have been developed. Genotyping by shallow survey sequencing (0.05–0.1×) in the model species rice (*Oryza sativa*) has been shown to yield genetic maps of unprecedented density (Chandler and Brendel, 2002; Xie *et al*., 2010). However, the high resolution of recombination breakpoints (approximately 40 kb) was provided by inferring marker order from a high-quality reference sequence. This approach cannot be applied to species with genomes of draft or even pre-draft quality as sequence contigs are not organized in pseudo-molecules representing the linear chromosomes.

The question of how several millions of markers provided by NGS technology may be used to bring contigs into a linear order (a procedure commonly referred to as anchoring) has only tentatively been raised. Andolfatto *et al*. (2011) used digestion with a frequently cutting restriction enzyme and subsequent multiplexed sequencing of a population of 94 individuals to assign 8 Mb of unassembled contigs to linkage groups. Similarly, a reduced-representation genotyping-by-sequencing method (Poland *et al*., 2012) has been instrumental in anchoring the barley physical map to a genetic map (International Barley Genome Sequencing Consortium, 2012). However, genotyping by WGS has not been used as a primary tool in *de novo* development of linearly ordered draft genome assemblies.

**Figure 1:**
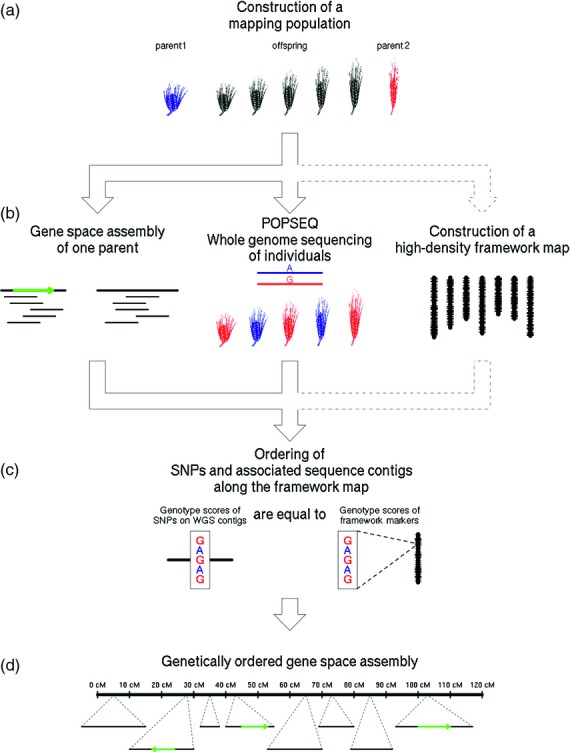
Schematic representation of POPSEQ.(a) A segregating population (80–100 individuals) is constructed from a bi-parental cross.(b) A whole-genome shotgun is generated for one parent, and used to construct a gene space assembly (alternatively, the POPSEQ data itself may be used for this purpose). On this assembly, gene models (green arrows) are defined using RNA–seq. In parallel, POPSEQ, and, if necessary, genotyping-by-sequencing (GBS), is performed on the population, and a medium-density framework genetic map is calculated (thousands to tens of thousands of loci).(c) SNPs detected and typed by POPSEQ along with associated WGS contigs are integrated into the framework map through nearest-neighbor search.(d) The result of POPSEQ is a sequence assembly in linear order that contains comprehensive information on the gene space. It may be enhanced by performing POPSEQ on additional populations.

In the absence of an appropriate molecular or analytical method to establish short-range connectivity (i.e. to link physically close sequence contigs), we used the power of genetic segregation to directly and linearly arrange sequence contigs into closely associated recombination bins along a target genome. We show that whole-genome survey sequencing of a small experimental segregating population and genetic mapping of the millions of observed single nucleotide polymorphisms (SNPs) detected therein (Figure 1) vastly improves the quality and utility of highly fragmented NGS shotgun assemblies. We illustrate the approach using the complex 5.1 Gb genome of cultivated barley (*Hordeum vulgare* L.) by comparing the output with a gene space assembly that has been partially ordered using extensive physical and genetic mapping resources (International Barley Genome Sequencing Consortium, 2012). Our results are congruent with the current sequence assembly (International Barley Genome Sequencing Consortium, 2012) but increase the amount of genetically anchored contig sequences by a factor of three. Most importantly, the whole effort cost <$100K and was completed in a matter of months. This new assembly has greater value for comparative genetic studies, gene isolation and genomics-assisted breeding compared to the previous anchoring effort (International Barley Genome Sequencing Consortium, 2012) as more WGS contigs are positioned genetically. In principle, the approach, which we term POPSEQ, may be used for any species for which a segregating population may be derived and maintained.

## Results

### Whole-genome survey sequencing of genetic populations

We generated survey sequences from 90 individuals (Table 1) of a population of recombinant inbred lines (RILs) from a cross between barley cultivars Morex and Barke (M × B). DNA from individual plants was fragmented and bar-coded, and eight samples per lane were sequenced on an Illumina HiSeq 2000 instrument (yielding approximately 1× coverage per line). We de-convoluted and mapped the output reads against a 50× WGS sequence assembly of the barley cultivar Morex (International Barley Genome Sequencing Consortium, 2012) using BWA software (Li and Durbin, 2009), and performed *in silico* variant calling using SAMtools (Li, 2011) (see Experimental procedures). This resulted in a set of SNP positions on the Morex WGS assembly, and genotype calls (i.e. homozygous for one parent or heterozygous) for each individual at each SNP. After discarding variant positions with low quality or too much missing data (Figure S1), 5.1 million SNPs with a mean of 33 unambiguous genotypic calls across the population were considered for integration into a high-density SNP-based genetic map of the same population constructed by array-based genotyping (Comadran *et al*., 2012). We then used a heuristic algorithm to place the newly discovered SNPs into this existing genetic framework. Briefly, we performed a nearest-neighbor search, querying the set of framework markers for elements with minimal Hamming distance to a given SNP (i.e. the minimum number of alternative SNP alleles required to change an observed segregation pattern into the reference) If several framework markers exhibited identical minimal distances, we imposed a cutoff where =80% of the framework markers had to lie on the same chromosome and the median absolute deviation of their genetic positions was less than five centiMorgans (cM). Using these thresholds 4.3 million SNPs (85.5% of all detected SNPs) could be placed into the genetic map with less than two genotype calls differing from their closest framework marker.

**Table 1:** Sequence data generated in this study

	M x B WGS	OWB WGS	M × B GBS	Morex
Population	Morex × Barke RIL F_8_	Oregon Wolfe Barleys DH	Morex × Barke RIL F_8_	–
Sequencing technology	Whole-genome shotgun; HiSeq 2000	Whole-genome shotgun; HiSeq 2000	Genotyping-by-sequencing; HiSeq 2000	Whole-genome shotgun; HiSeq 2000
Number of sequencing lanes	12	12	1	2
Number of sequenced individuals	90 (+ parents)	82 (+ parents)	92 (+ parents)	1
Approximate coverage per sample	1×	1×	1× (10 Mb represented)	15×
Number of SNPs detected	5 123 696	6 543 684	21 397	–
Mean number of present genotype calls per marker	33	31	58	–

We then assigned the WGS sequence contigs that harbored mapped polymorphisms to their defined genetic positions. As with positioning SNPs in a genetic map, we imposed a rule that multiple SNPs found on the same sequence contig were required to have concordant genetic positions. Overall, 498 856 contigs with a cumulative length of 927 Mb (49.5% of the total cv Morex WGS sequence assembly) could be ordered along the genetic map (Table2), more than doubling the 410 Mb that was anchored with the help of a genome-wide physical map to the same genetic framework. Tables containing the anchoring results are available for download from ftp://ftp.ipk-gatersleben.de/barley-popseq/

**Table 2:** Anchoring statistics

	M x B (iSelect)[Table-fn tf2-1]	OWB	M x B (GBS map)	M x B + OWB	IBSC
Number of SNPs used for anchoring	4 381 020	6 117 837	4 429 475	11 229 709	498 165
Framework map	iSelect	OWB GBS	M x B GBS	iSelect/OWB GBS	iSelect
Number of anchored contigs	498 856	591 779	512 293	747 077	138 443
Size of anchored contigs (Mb)	927 (50%)	1000 (53%)	934 (50%)	1222 (65%)	410 (16%)
Median length of anchored contigs (bp)	1006	973	977	891	1431
Number of anchored HC genes[Table-fn tf2-2]	16 682 (64%)	15 743 (60%)	16 729 (64%)	20 932 (80%)	15 719 (60%)
Number of anchored LC genes[Table-fn tf2-3]	28 337 (56%)	29 033 (55%)	28 559 (56%)	37 609 (71%)	19 415 (36%)

The Morex × Barke iSelect framework map is described in International Barley Genome Sequencing Consortium (2012) and Comadran *et al*. (2012).

High-confidence genes as described in International Barley Genome Sequencing Consortium (2012).

Low-confidence genes as described in International Barley Genome Sequencing Consortium (2012).

### Validation of population sequencing

We checked whether the genetic anchoring generated by POPSEQ was consistent with available short-range connectivity information. The (International Barley Genome Sequencing Consortium, 2012) had sequenced 6278 bacterial artificial chromosomes (BACs). Individuals BACs were sequenced to ‘Phase 1 quality and consisted on average of five to ten sequence contigs. From this set, we identified 3902 clones that harbored at least two WGS contigs that were mapped by POPSEQ. Our hypothesis was that in the majority of cases, pairs of contigs from the same BAC clone (i.e. within a physical distance of less than 200 kb) would exhibit the same genetic location. Using ultra-stringent homology (100% identity over 1000 bp), 95% of the contig pairs were placed within a 3 cM window on the ordered assembly (Table S1). Discordant chromosome assignments were found for only 1.7% of the contig pairs, and a further 3.3% had a genetic distance larger than 3 cM. We inspected 17 BACs with at least five anchored WGS contigs and discordant chromosome assignments. Nine of these BACs had two groups of contigs anchored to different locations and had either suspiciously large insert sizes of =180 kb suggestive of chimeric inserts or showed evidence of independent clones having been sequenced under the same name.

We then compared the POPSEQ anchoring of WGS contigs to a recently released integrated sequence-enriched genetic and physical map of barley (International Barley Genome Sequencing Consortium, 2012). More than 77 000 WGS contigs (representing 315 Mb of sequence) were assigned by both methods to specific genetic positions. Chromosome assignments disagreed in 2.2% of the cases, and cM coordinates differed by more than 5 cM in 7.0% of the cases, similar to the 2–8% false-positive rate observed in PCR-based screening of BAC libraries (International Barley Genome Sequencing Consortium, 2012). In general terms, incongruence appears to occur largely in the highly repetitive and extensive genetic centromeres. We believe that this is most likely the product of misplaced repetitive sequence-containing or chimeric BAC contigs in the barley physical map. Thus, employing POPSEQ alongside a fully sequenced minimum tiling path highlights errors in a physical map and its associated anchoring information, and may thereby be valuable in establishing a robust clone-by-clone assembly of a target genome.

### Framework map construction by GBS

**Figure 2:**
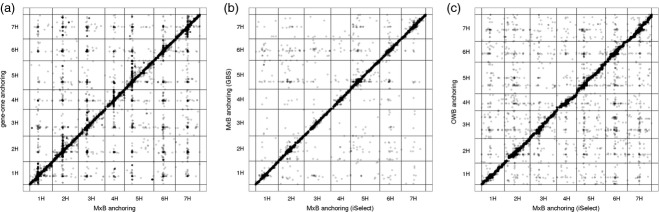
POPSEQ validation. WGS contigs anchored to three genetic maps. These plots show the colinearity of contigs anchored to the Morex × Barke iSelect framework map and (a) the physical and genetic framework of barley (International Barley Genome Sequencing Consortium, 2012), (b) a Morex × Barke genetic map constructed by genotyping-by-sequencing (GBS), (c) a GBS map (Poland *et al*., 2012) constructed in the OWB. WGS contigs are shown as dots, and are mostly within 5 cM of the diagonal: 90.8% in (a), 99.2% in (b) 93.2% in (c).

To further investigate the robustness of POPSEQ, we assessed the effect of using a different genotyping platform to construct the framework map. We genotyped the same 90 individuals using a two-enzyme genotyping-by-sequencing (GBS) approach (Poland *et al*., 2012) (Table1). Prior to sequencing, DNA was digested using a rarely and a frequently cutting restriction enzyme, and only restriction fragments with two different restriction sites were sequenced, thus reducing the targeted interval on the genome to approximately 10 Mb. Compared to array-based genotyping, GBS has lower per-sample costs and does not require any prior knowledge of polymorphisms between the parents of the population. Instead, marker detection and scoring occur simultaneously, making GBS suitable for species without any genomic resources, or for which genomic resources are poorly developed. We constructed a *de novo* genetic map comprising 4056 bi-allelic SNP markers, and placed WGS contigs into this map using the same algorithm as described above. Altogether, 927 Mb of sequence represented by 512 293 sequence contigs was ordered (Table 2), with 94.3% also linked to the iSelect framework (Comadran *et al*., 2012). Importantly, the genetic coordinates of contigs were consistent among the underlying framework maps (Figure 2b): chromosome assignments were discordant in 0.1% of the cases, and the map position of only 0.6% of the contigs differed by more than 5 cM. If we only used the SNP markers (approximately 20 000) provided by GBS, we were able to anchor only 49 Mb of sequence, because the number of anchored contigs is limited by the number of available SNPs.

### Robustness of the linear assembly

To test the robustness of the M x B POPSEQ anchored assembly, we constructed a *de novo* assembly of a second population for comparison. We used the Oregon Wolfe Barley (OWB) population, as a genetic map from GBS on 82 doubled haploid (DH) lines was already available (Poland *et al*., 2012). We survey-sequenced these 82 individuals to approximately 1× whole genome coverage each (Table 1), and, by performing the same steps as for M x B, assigned genetic positions to 591 779 WGS contigs corresponding to 1000 Mb of sequence. Of these contigs, 42% (295 Mb) were not anchored to the M x B iSelect framework. In most cases, these contigs either harbored no polymorphism between Morex and Barke, or SNPs were not assayed in a sufficient number of RILs to reach our threshold for inclusion. Contigs anchored to both M x B and OWB maps had highly congruent chromosome assignments (99.6% agreement, Figure 2c). Only 6.4% of all contigs were placed more than 5 cM apart in the two anchored assemblies (falling to 2.1% if <7 cM). Given that we were comparing populations constructed with different parents and levels of recombination (approximately half in a DH population compared to RILs), this was not completely unexpected. However, the use of independent populations for anchoring has considerable value: the cumulative length of contigs anchored to either the M x B or OWB map is 1.22 Gb, an increase of one-third compared to use of only a single population. Additional polymorphisms in OWB thus enabled placement of contigs that were identical between Morex and Barke. More importantly, the POPSEQ ordered assembly positions an additional 5213 annotated high-confidence genes on the barley genome compared to the International Barley Genome Sequencing Consortium release.

### Framework map construction using light shotgun population sequencing

We then explored whether the POPSEQ data could be used directly to construct a robust *de novo* genetic map without reference to other datasets or genotyping methods. Briefly, we identified a set of 65 357 contigs containing at least ten Morex/Barke SNPs per contig, requiring that these contigs be genotyped by shallow WGS sequencing in at least 75 of the 90 individuals within our M × B mapping population to avoid an excess of missing data points. Using stringent controls on log-odds scores, 98.5% of these contigs were readily clustered into seven major linkage groups and ordered by MSTMap (Wu *et al*., 2008). The resulting framework map has approximately 99% concordance with existing barley maps (Pearson correlation coefficient), and may be used to place additional contigs with fewer SNPs and/or more limited sampling using a majority rule approach as described above. Thus POPSEQ data may be used directly to generate a linear ordering of contigs, even in the absence of an independent genetic map.

### POPSEQ does not require long mate-pair libraries

The set of whole-genome contigs (the ‘reference assembly’) used in the present study had been assembled from Illumina libraries with fragment sizes of 350 bp and 2.5 kb (International Barley Genome Sequencing Consortium, 2012). Although large-insert mate-pair libraries may be used to establish links between contigs, and may be required input for some assemblers (Gnerre *et al*., 2011), the construction of such libraries is not straightforward and often yields sub-optimal results, such as a high fraction of PCR duplicates or short-insert read pairs. We therefore explored how POPSEQ performed using an assembly comprising only short-insert paired reads. We sequenced the same 350 bp insert libraries used for construction of the current barley reference assembly (International Barley Genome Sequencing Consortium, 2012) on two HiSeq lanes, yielding approximately 15× haploid genome coverage (Table1), and assembled the reads using the same program as previously (International Barley Genome Sequencing Consortium, 2012). As the read coverage was approximately three times lower than used by International Barley Genome Sequencing Consortium (2012) and did not utilize mate-pair information, we expected the assembly to be of worse quality. The cumulative length of the resulting assembly was shorter (1.6 Gb versus 1.9 Gb), and the contig N50 (a weighted average contig size that is commonly used as a measure of assembly contiguity) was smaller (1238 bp versus 1450 bp). However, contigs of this size are sufficient to function as a reference for read mapping and to enable structural gene annotation via RNA sequencing (RNA-seq) as well as SNP detection. Notably, almost half of the contigs (49.8%) anchored to the M x B iSelect framework are shorter than 1000 bp. In species with smaller and less repetitive genomes, WGS assembly is expected to yield fewer and longer contigs that potentially yield a higher number of SNPs per contig (depending upon the level of polymorphism in the POPSEQ population). Alternatively, larger contigs may compensate for lower levels of polymorphism.

## Discussion

Low-coverage (approximately 0.05–0.1×) NGS survey sequencing of the small genome (0.4 Gb) of the model crop plant rice has previously been used as a tool to generate many thousands of genetic markers for both bi-parental linkage studies and GWAS (Huang *et al*., 2009, 2010). The effectiveness of this ‘genotyping by re-sequencing’ was afforded by the availability of high-quality reference sequences, a small target genome with comparatively few repeats, and innovative statistical approaches to data analysis. Here, we have explored a fundamentally different application of NGS combined with classical genetic analysis that should find application in many species, particularly those with recalcitrant, large or poorly characterized genomes, among them economically important species such as wheat, sugarcane, pine or *Miscanthus*.

We explored POPSEQ as a method for genetically anchoring and ordering *de novo* NGS assemblies, and have demonstrated its potential by re-synthesizing and improving a recently released sequence assembly of the large (5.1 Gb) and complex (= 80% repetitive sequence, ancestrally duplicated) barley genome. We used sequence data from two mapping populations, and used the large number of detected SNPs to integrate the sequence assembly with two established framework maps as well as genetic maps computed from GBS or WGS data. At its core, POPSEQ exploits the power of genetic segregation combined with shallow (1–2× per line) survey sequencing of one or more small experimental populations to genetically anchor NGS sequence assemblies. It is independent of physical mapping and all other genomic resources typically developed in large genome sequencing projects, and should be amenable to application in most population types.

We show that POPSEQ is both robust and reproducible. Using various genetic maps and mapping population, we obtain comparable results with a concordance of approximately 95%. Thus, POPSEQ is neither dependent upon the choice of mapping population nor the genotyping platform used for framework map construction. If more extensive short-range connectivity is established by longer sequence contigs or scaffolds (set of ordered sequence contigs with gaps between them), a sliding window approach (Huang *et al*., 2009) may be used for genotype calling and framework map construction from POPSEQ data alone, avoiding the need for GBS or SNP mapping platforms. In addition, partitioning of polymorphic sites according to their parental origin may be performed prior to *de novo* assembly, for example by using the colored de Bruijn graph method (Iqbal *et al*., 2012). The raw sequence reads from POPSEQ (the equivalent of 50× for each parent) should then be sufficient to compute the reference sequence assemblies that will ultimately be ordered along the genetic map.

POPSEQ performs effectively with highly fragmented sequence assemblies from short-insert libraries. We were able to construct a *de novo* WGS assembly from short Illumina reads that showed assembly statistics comparable to an assembly that incorporated mate-pair information. POPSEQ thus avoids the technical difficulties associated with construction and characterization of large-insert libraries. The simultaneous use of several mapping populations through sequence-based consensus map construction is straightforward, with the same caveats as observed in any genetic map integration. The outcome is not merely an ultra-dense genetic map of anonymous loci: at each genetic position, comprehensive information on the gene space may be obtained through RNA–seq-based structural annotation.

The POPSEQ resource we developed here both reproduces and substantially improves the multi-layered gene space assembly that was the result of a large collaborative effort by the International Barley Genome Sequencing Consortium over many years. By comparison, POPSEQ is inexpensive, rapid and conceptually simple, the most time-consuming step being the construction of a mapping population. In relation to the latter, while we used both DH lines and RILs, other population types including early-generation inbred lines (e.g. F_4_ individuals) would also be suitable. Subsequent steps including sequence assembly from short-insert libraries, genotyping-by-sequencing (if required) and integrative computational analyses may be performed quickly. We stress that we do not advocate abandonment of on-going genome projects that are pursuing a clone-by-clone strategy. On the contrary, we believe these may profit from POPSEQ. BAC contigs may be validated though genetic mapping of each single clone, and the high number of mapped genetic markers should allow virtually any fully sequenced physical contig to be accurately placed.

Having performed a proof of principle in barley, the notion of advancing the closely related bread wheat genome (Paux *et al*., 2008) by adopting POPSEQ is of particular interest. Wheat will be the last of the world’s major crops to be fully sequenced. The cost-efficient construction of high-density genetic maps is routine in hexaploid wheat (Poland *et al*., 2012), and the challenge of distinguishing homoeologous sequences has been largely overcome: sub-genome-specific shotgun assemblies have been released recently (Brenchley *et al*., 2012), and chromosome-specific survey sequences have also been generated (Hernandez *et al*., 2012). Furthermore, several populations of recombinant inbred lines are already available within the academic and commercial sectors, and are ripe for exploitation (Nelson *et al*., 1995; Manickavelu *et al*., 2011). While the ultimate goal should be a clone-by-clone sequence of the wheat genome with a quality on par with that of the the maize genome, POPSEQ opens the way to obtain, with comparative ease, an effective surrogate that would be valuable for basic research and breeding applications. In addition to wheat, many non-model species, orphan crops and old genetic models such as pea (*Pisum sativum*), have not yet benefited much from the genomics era. With moderate effort, POPSEQ could allow the generation of highly useful sequence resources for these and many other species.

For an uncharacterized =5 Gb diploid genome, between 14 and 30 HiSeq lanes are required for (i) producing a *de novo* sequence assembly for read mapping (two to eight lanes; not required if POPSEQ data itself is used to produce the ‘reference’ sequence assembly); (ii) genotyping-by-sequencing for map construction (one lane; not required if POPSEQ is used to construct the reference map); (iii) shallow population sequencing (minimum 12 lanes for each population of approximately 90 lines, although the depth may be varied); (iv) deep RNA-seq for structural gene annotation (more than two lanes) amounting to $50 000–$100 000 in sequencing costs. Together with a medium-sized computer server (32 CPU cores, 512 GB RAM, 3 TB of disk space), it is possible to generate a *de novo* linear gene space assembly.

The accuracy of POPSEQ may be improved if the members of the population are sequenced to higher depth. With the sequencing depth used in this study (1–2×), the sequencing reads of each individual cover only approximately 50% of the assembly. Doubling the amount of sequencing data per individual would result in genome coverage of approximately 80% according to the model of Lander and Waterman (1988) (Figure S2), thus reducing the number of missing genotype calls per individual. An increase in sequencing depth is mandatory for highly heterozygous populations such as F_2_ populations in selfing organisms or F_1_ populations in outcrossing species in order to correctly type heterozygous SNPs. Using an improved assembly with longer contigs or contigs organized into physically close scaffolds would benefit the analysis, as more SNPs could be used to place each sequence contig. An increase in the number of sequenced individuals (resulting in a proportional increase in the sequencing load) may improve the genetic resolution of the framework map.

We propose that POPSEQ may contribute substantially to fundamental research in plant genetics as well as in crop improvement (for examples, see Figure S3, Appendix S1 and Methods S1). However, its application is not restricted to plants. The fast and steady advances in sequencing technology will further increase the power of POPSEQ, allowing deeper coverage of larger and outbred populations. As long as the inherent complexity of genomes restricts the assembly of pseudo-molecules by shotgun sequencing, POPSEQ provides a rapid, low-cost and effective method for developing a highly useful ‘interim reference’ genome sequence in most species for which it is possible to construct a genetic map.

## Experimental procedures

### Whole-genome shotgun sequencing

Illumina paired-end libraries(fragment size approximately 350 bp) were generated from fragmented genomic DNA of 90 individuals from the Morex × Barke RIL population and 82 individuals of the OWB population. Individual libraries were bar-coded prior to combining in pools of eight and sequencing on Illumina HiSeq 2000 instruments (http://www.illumina.com). The iSelect framework was available from a previous study (Comadran *et al*., 2012).

### Read mapping and SNP calling

Sequencing reads were quality trimmed and mapped against the Morex WGS assembly (International Barley Genome Sequencing Consortium, 2012) using BWA version 0.6.2 (Li and Durbin, 2009). The command ‘bwa aln’ was used with the parameter ‘-q 15’ for quality trimming, otherwise default parameters were used. After removing duplicate reads using the SAMtools (Li, 2011) command rmdup, variant positions and genotypes of individuals at variant positions were called using the SAMtools mpileup/bcftools pipeline version 0.1.18 (Li, 2011) with default parameters. Additionally, the parameter ‘-D’ was used for SAMtools mpileup to record per-sample read depth. The resulting VCF file was filtered using a custom AWK script. The script removed SNPs with a SAMtools quality score below 40, and further filtered SAMtools genotype calls: a homozygous genotype call was retained if there was at least one read supporting it and its SAMtools genotype quality was at least 3. In the M x B data, a heterozygous call was retained if there were at least three supporting reads and its score was at least 5. In the OWB DH population, heterozygous calls were always discarded. Genotype calls not matching the specified criteria were set to a missing value. A variant position was removed if more than 10% of all samples were called heterozygous, there were more than 80% missing data, or the minor allele frequency (in the non-missing data) was smaller than 5%.

### Mapping SNPs and WGS contigs to the framework map

The nearest neighbors of SNPs detected in the WGS shotgun data were searched for using a heuristic algorithm implemented in GNU C. The source code is available from ftp://ftp.ipk-gatersleben.de/barley-popseq/.As a metric, we used the minimum Hamming distance. The nearest neighbors were searched for in the set of (i) 1723 non-redundant iSelect SNPs, (ii) 4056 GBS SNPs used for construction of the M x B GBS map, and (iii) 4632 non-redundant OWB GBS SNPs meeting these criteria described below. A SNP was considered redundant if there was another SNP with the same genotype (on the non-missing data) and the same genetic position. +Correction added on 25 October 2013 after original online publicationon 10 October 2013: ftp://ftp.gatersleben.de/barley-popseq/was changed to ftp://ftp.ipk-gatersleben.de/barley-popseq/-

SNPs were used to anchor WGS contigs if they were scored unequivocally on more than 20% of the individuals in the population, the Hamming distance (number of different, non-missing genotypes) to their nearest SNPs was not larger than 2, at least 80% of all nearest SNPs lay on the same chromosome, and the median absolute deviation of the cM positions (on the chromosome with most markers) was <5 for the OWB map and the M x B iSelect framework. As we used the population type DH for the M x B RILs (as required for advanced RILs), the M x B GBS map over-estimated the map length by a factor of approximately 3, and we allowed a maximal median absolute deviation of 15. The cM coordinate of a SNP meeting these criteria was defined as the median cM position of its nearest neighbors. A WGS contig was assigned to a genetic position if at least 80% of all SNPs located on it had been mapped to the same chromosome and the median absolute deviation of the cM coordinates of the SNPs was <5 (15 for M × B GBS). The cM position of a contig was set to the median cM position of all SNPs located on the contig.

### Estimation of the error rate

WGS contigs were compared using MegaBLAST version 2.2.26 (Zhang *et al*., 2000) to 6278 fully sequenced BACs. Under stringent criteria, we required 100% identity and a minimum alignment length of 1000 bp for each BLAST High Scoring Pair. Under relaxed criteria, we required 99% identity and 200 bp minimum alignment length. The genetic positions of all pairs of contigs on the same BAC were compared (Table S1). BACs with discordant chromosome assignments and with hits to at least five anchored contigs were further analyzed. For each BAC, the chromosome assignments of its contigs were tabulated. If at least 30% of all contigs on a BAC were anchored to the chromosome with the second highest number of contigs, the BAC was deemed problematic, and we checked whether it had been sequenced twice or its length (the cumulative length of its assembled sequence contigs) was unusually large (=180 kb).

### Genetic map construction from M x B GBS data

GBS library production and sequencing for M x B populations were performed as described previously (Poland *et al*., 2012). Reads were deconvoluted using a custom AWK script. Adapter sequences were removed using cutadapt version 1.1 (http://code.google.com/p/cutadapt). Trimmed reads shorter than 30 bp were discarded. Read mapping, SNP and genotype calling, and filtering were performed essentially as described above for the WGS data. As only single ends were used, the BWA command samse was used for alignment. Additionally, only SNPs meeting the following criteria were considered for genetic map construction: no more than 10% missing data, no more than 10% heterozygous genotypes, 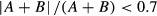, where *A* and *B* indicate the counts of the parental alleles; in the absence of heterozygous calls, this corresponds to a minimum minor allele frequency of 17.6%. For M × B, 4058 SNPs passed these filters. Genetic map construction was performed using MSTMap (Wu *et al*., 2008) with the following parameters: population type DH; distance function kosambi; cut_off_p_value 0.00001; no_map_dist 20; no_map_size 2; missing_threshold 0.8; estimation_before_clustering no; detect_bad_data yes; objective_function COUNT. The resulting map contained seven linkage groups with more than one marker. Two markers formed a linkage group of their own and were discarded. According to the obtained orders, orientations and distances between markers, the linkage groups corresponded to the seven barley chromosomes. The relationship between genetic positions in the new map and the iSelect map was obtained through Loess regression +R (http://www.r-project.org) function loess, smoother span 0.3-. Interpolation into the iSelect map of WGS SNP positions integrated to the GBS framework was performed using the loess model with the R function predict.

### De novo *map construction from POPSEQ*

To build an independent genetic map from the POPSEQ data without reference to existing maps or other marker data, we restricted our attention to the 115 258 sequence contigs that span at least ten SNPs that are polymorphic between the two parents Morex and Barke. For the purposes of developing a framework map, we further restricted our attention to contigs with highly concordant SNP genotype calls. We therefore set aside contigs that had two or more SNP genotype calls from both parents, indicating the possibility of mis-genotyping through incorrect SNP calls and/or limited cross-contamination between individuals. The resulting 80 189 contigs were then genotyped as either Morex or Barke based on the consensus of their genotyped SNPs, requiring at least three SNP calls. Finally, for the framework map, we only considered contigs that could be consensus genotyped in at least 75 of the 90 individuals. This left us with 66 357 contigs that could be reliably genotyped with limited missing data. We computed the recombination rate and logarithm of odds (LOD) score between each pair of contigs, and clustered contigs with LOD = 10 to form linkage groups: 64 476/65 357 (98.7%) of contigs formed 14 linkage groups, with approximately 98.87% of contig length placed in seven major linkage groups, corresponding to the seven barley chromosomes.

### Integration of WGS SNPs to the OWB GBS bin map

A bin map (Poland *et al*., 2012) had previously been constructed from GBS data of 82 OWB DH lines. GBS marker sequences (64 bp long) were aligned against the Morex WGS assembly using the BWA command ‘aln’ and the command ‘samse’. Only alignments with the best possible mapping score of 37 were considered. SNPs with missing data for the parents or more than 10% missing data on the DH lines were not considered for the nearest-neighbor search. The anchoring of SNPs and contigs has been described above.

### *De novo* assembly

Illumina paired-end libraries (insert size 350 bp) for barley cultivar Morex had been constructed previously (International Barley Genome Sequencing Consortium, 2012). Sequencing on the Illumina HiSeq 2000 was performed according to the manufacturer’s procedures (http://www.illumina.com). Sequencing reads were quality trimmed and assembled using CLC assembly cell 3.2.2 (http://www.clcbio.com).

## Accession Numbers

The accession numbers for the sequences described are ERP002183 (GBS sequence data of the Morex × Barke RILs) and ERP002184 (whole-genome shotgun sequence data for the Morex × Barke RILs and OWB DH lines).

## Supporting Information

Figure S1. Distribution of the number of successful genotype calls at variant positions detected in the whole data of the Morex x Barke and OWB populations.

Figure S2. Observed and expected sequence coverage according to the model of Lander and Waterman (1988).

Figure S3. Potential uses for an assembly ordered by POPSEQ.

Table S1. The percentage of WGS contigs pairs assigned to the same BAC that are positioned farther apart than the specified distance.

Appendix S1. Applications of a POPSEQ assembly for comparative genomics, reference-based genetic mapping and gene isolation.

Methods S1. Experimental procedures for Appendix S1.
