# Supplementary material for: Anchoring and ordering NGS contig assemblies by population sequencing (POPSEQ)
Source: Plant J. 2013 Oct 10;76(4):718–27. doi: 10.1111/tpj.12319 (PMC4298792; doi:10.1111/tpj.12319)
Supplement: Supplementary file 1 [file tpj0076-0718-sd1.docx]

**Mascher *et al* Supporting Figure Legends**

**Figure S1:**

This graphic shows the distribution of the number of successful genotype calls at variant positions detected in the whole data of the Morex x Barke and OWB populations. Variant positions with more than 80 % missing data were not used for downstream analysis.

**Figure S2:**

Potential uses for an assembly ordered by POPSEQ. The graphic is described in Appendix S1.

**Figure S3:**

Observed and expected sequence coverage according to the model of Lander and Waterman.

The number of reference base that is covered by at least one sequence read. Each dot represents the sequencing data from one individuals of Morex x Barke population. The red line is the theoretical genome coverage (given the sequencing output and the expected genome size) according to [Lander and Waterman (1988](#_ENREF_5)). The expected coverage according to the original formula was multiplied by a factor of 0.9 to fit the observed values more closely. The original formula overestimated the genome coverage, most likely because it did not take unmappable reads into account.

**Supporting Reference**

Lander ES, Waterman MS. 1988. Genomic mapping by fingerprinting random clones: a mathematical analysis. *Genomics* **2**(3): 231-239.
