# Supplementary figures and images for: Anchoring and ordering NGS contig assemblies by population sequencing (POPSEQ)
Source: Plant J. 2013 Oct 10;76(4):718–27. doi: 10.1111/tpj.12319 (PMC4298792; doi:10.1111/tpj.12319)

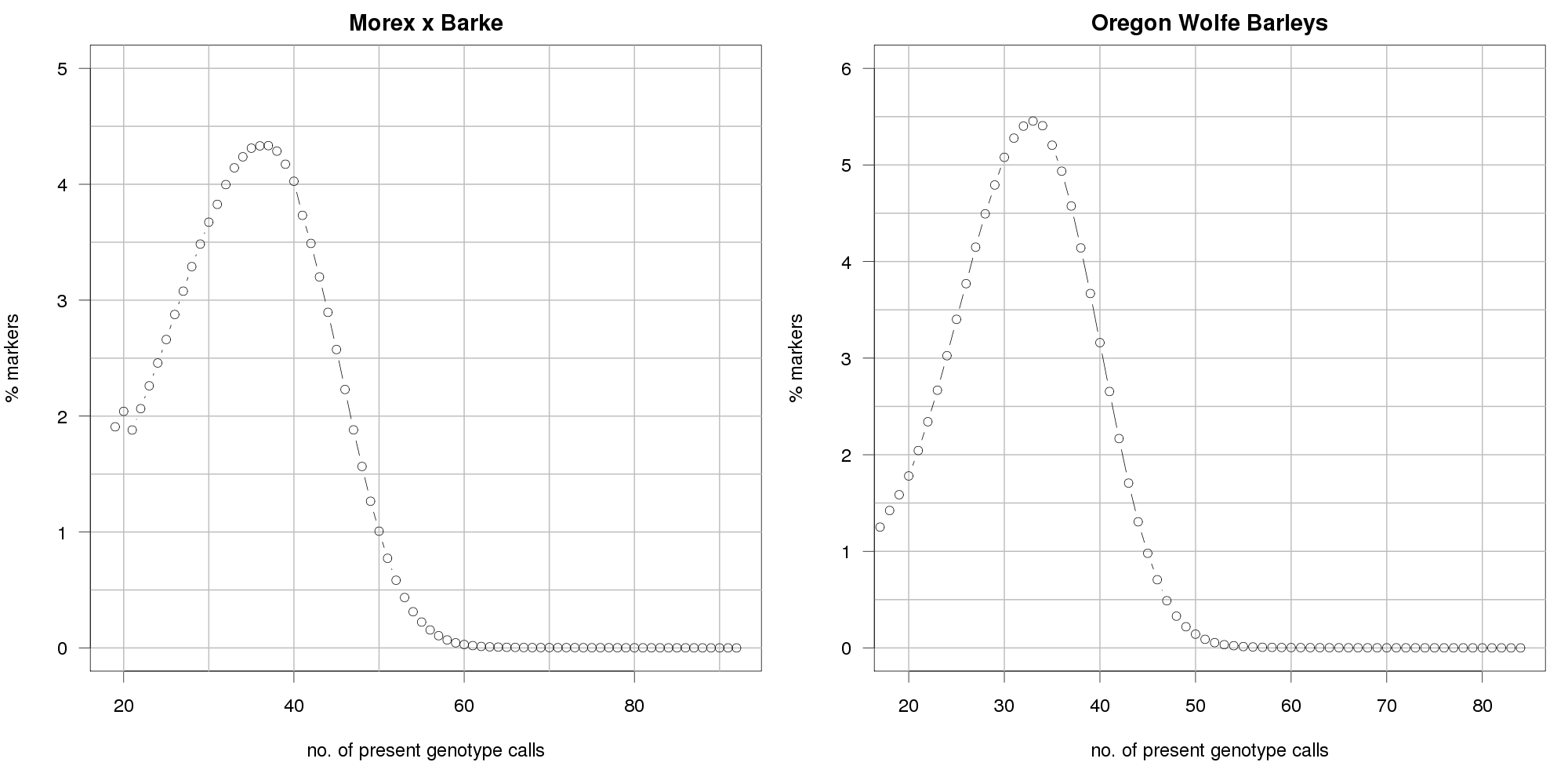

Supplement: Supplementary file 2 — Figure S1. Distribution of the number of successful genotype calls at variant positions detected in the whole data of the Morex x Barke and OWB populations. [file tpj0076-0718-sd2.png]

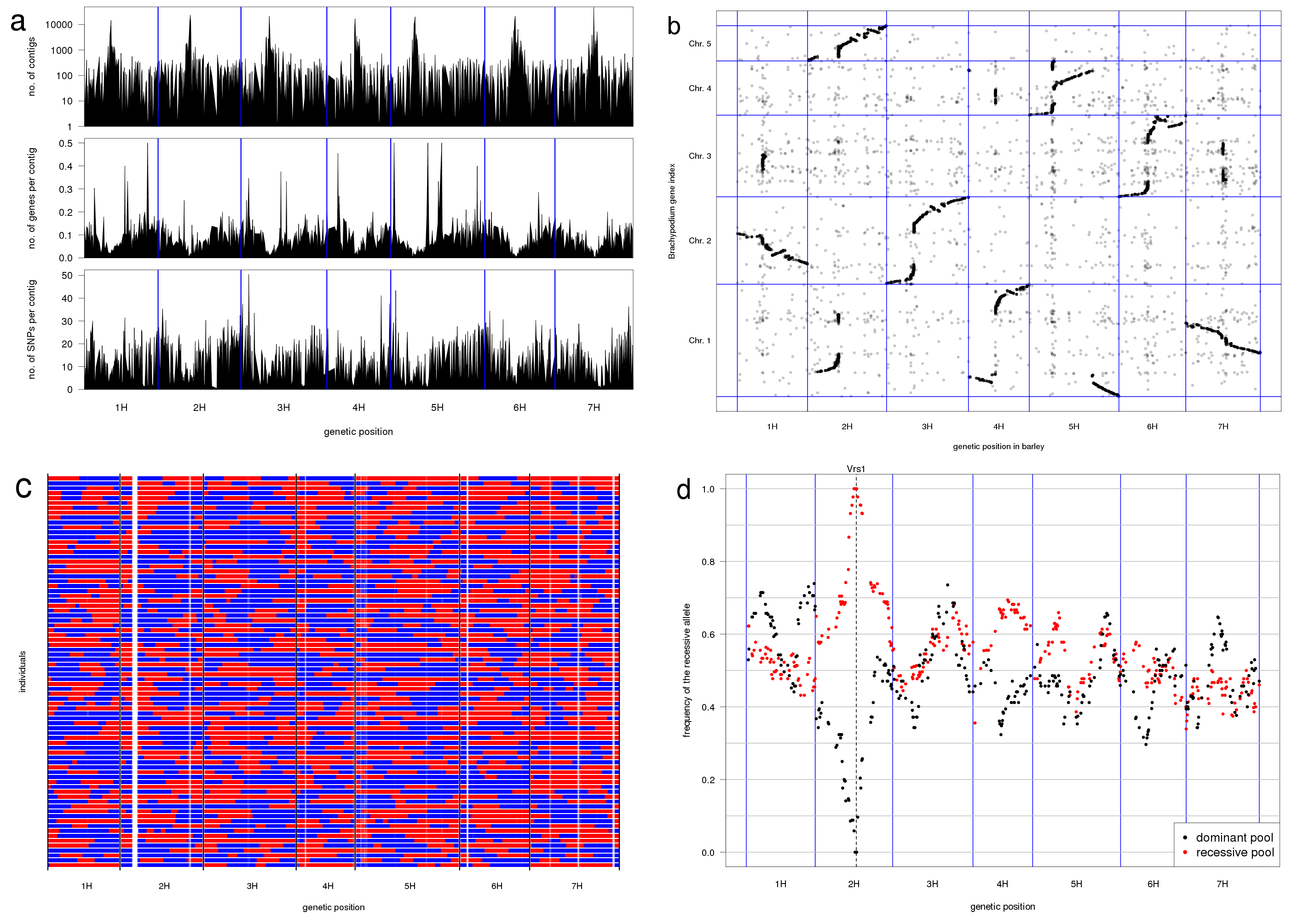

Supplement: Supplementary file 3 — Figure S2. Observed and expected sequence coverage according to the model of Lander and Waterman (1988). [file tpj0076-0718-sd3.png]

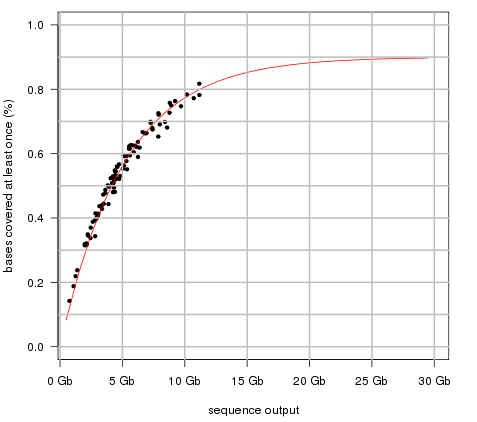

Supplement: Supplementary file 4 — Figure S3. Potential uses for an assembly ordered by POPSEQ. [file tpj0076-0718-sd4.png]
