# Supplementary material for: Anchoring and ordering NGS contig assemblies by population sequencing (POPSEQ)
Source: Plant J. 2013 Oct 10;76(4):718–27. doi: 10.1111/tpj.12319 (PMC4298792; doi:10.1111/tpj.12319)
Supplement: Supplementary file 5 — Table S1. The percentage of WGS contigs pairs assigned to the same BAC that are positioned farther apart than the specified distance. [file tpj0076-0718-sd5.docx]

**Mascher *et al* Supporting Table S1**

**Table S1:**

| Distance | MxB WGS (iSelect) | MxB WGS (GBS map) | OWB |
| --- | --- | --- | --- |
| > 0.5 cM | 29.28% | 29.61% | 35.40% |
| > 1 cM | 14.97% | 16.19% | 21.95% |
| > 1.5 cM | 8.86% | 9.32% | 20.86% |
| > 2 cM | 5.83% | 6.00% | 15.99% |
| > 2.5 cM | 4.38% | 3.21% | 15.58% |
| > 3 cM | 3.25% | 2.23% | 11.73% |
| > 3.5 cM | 2.48% | 1.79% | 11.48% |
| > 4 cM | 1.86% | 1.45% | 8.42% |
| > 4.5 cM | 1.45% | 1.25% | 8.12% |
| > 5 cM | 0.99% | 1.06% | 5.81% |
| > 5.5 cM | 0.88% | 0.93% | 5.68% |
| > 6 cM | 0.85% | 0.82% | 3.91% |
| > 6.5 cM | 0.77% | 0.77% | 3.79% |
| > 7 cM | 0.67% | 0.71% | 2.61% |
| > 7.5 cM | 0.61% | 0.69% | 2.54% |
| > 8 cM | 0.59% | 0.63% | 1.89% |
| > 8.5 cM | 0.55% | 0.59% | 1.88% |
| > 9 cM | 0.52% | 0.52% | 1.49% |
| > 9.5 cM | 0.45% | 0.49% | 1.47% |
| > 10 cM | 0.43% | 0.47% | 1.33% |
| different chromosome | 1.66% | 1.79% | 2.77% |

The table shows the percentage of WGS contigs pairs assigned to the same BAC that are positioned farther apart than the specified distance. WGS contigs were assigned to BACs under stringent BLAST criteria (alignment length >=1000, 100 % identity).
