# Supplementary material for: Anchoring and ordering NGS contig assemblies by population sequencing (POPSEQ)
Source: Plant J. 2013 Oct 10;76(4):718–27. doi: 10.1111/tpj.12319 (PMC4298792; doi:10.1111/tpj.12319)
Supplement: Supplementary file 6 — Appendix S1. Applications of a POPSEQ assembly for comparative genomics, reference-based genetic mapping and gene isolation. [file tpj0076-0718-sd6.docx]

**Mascher *et al* Appendix S1:**

An anchored assembly does not establish an immediate relationship between genetic and physical distance. By averaging in genetic bins (1 cM in size), it is, however, possible to appreciate the fact that the genetic centromeres of barley chromosome show restricted recombination, a lower gene density and a lower SNP frequency **(a)** as we observed earlier ([IBSC, 2012](#_ENREF_2)).

Through RNA-seq gene models can be defined even on highly fragmented assemblies. The linear order of the sequence contigs allowed us determine blocks of conserved synteny between barley and the model grass *Brachypodium distachyon* ([International Brachypodium Initiative 2010](#_ENREF_3)) by comparing the positions of orthologous genes in the two genomes **(b)**.

In presence of an ordered gene space assembly, the effort of constructing genetic maps need not be taken for every new population. Instead, reference-based genotyping is possible. Part **(c)** shows the graphical genotypes for 79 OWB lines. SNP markers obtained through GbS ([Poland et al. 2012](#_ENREF_7)) were positioned on the Morex WGS assembly by homology search and ordered along the MxB map (iSelect framework).

Lastly, we assessed the usefulness of a genetically ordered assembly for gene isolation. Mapping-by-sequencing works by inspecting the allele frequency distribution in a segregating population ([Schneeberger et al. 2009](#_ENREF_8)). It has been successfully applied in model species and, in an ideal setting, it can identify the genes underlying a specific phenotype in a single step. We tried to map the Vrs1 gene ([Komatsuda et al. 2007](#_ENREF_4)), responsible for a either six- or two-rowed phenotype, in the OWB population using only genotyping-by-sequencing data. We visualized the frequency of the dominant and recessive alleles along the genetic length of the chromosomes **(d).** A clear peak is visible on chromosome 2H at two consecutive bins (77 and 80 cM). No GBS markers were mapped in between. The interval contains 1,121 WGS sequence contigs (2.1 Mb) harboring 58 high confidence and 70 low confidence genes. The Vrs1 gene itself is annotated as a low confidence gene on a WGS contig anchored at 79.4 cM on 2H. Through further fine-mapping in a larger population, deeper sequencing or the analysis of differential gene expression, cloning of the causal gene would be possible.

**Supporting References**

IBSC. 2012. A physical, genetic and functional sequence assembly of the barley genome. *Nature* **491**(7426): 711-716.

International Brachypodium Initiative T. 2010. Genome sequencing and analysis of the model grass Brachypodium distachyon. *Nature* **463**(7282): 763-768.

Komatsuda T, Pourkheirandish M, He C, Azhaguvel P, Kanamori H, Perovic D, Stein N, Graner A, Wicker T, Tagiri A et al. 2007. Six-rowed barley originated from a mutation in a homeodomain-leucine zipper I-class homeobox gene. *Proceedings of the National Academy of Sciences of the United States of America* **104**(4): 1424-1429.

Poland JA, Brown PJ, Sorrells ME, Jannink JL. 2012. Development of high-density genetic maps for barley and wheat using a novel two-enzyme genotyping-by-sequencing approach. *PLoS One* **7**(2): e32253.

Schneeberger K, Ossowski S, Lanz C, Juul T, Petersen AH, Nielsen KL, Jorgensen JE, Weigel D, Andersen SU. 2009. SHOREmap: simultaneous mapping and mutation identification by deep sequencing. *Nature methods* **6**(8): 550-551.
