# Supplementary material for: Anchoring and ordering NGS contig assemblies by population sequencing (POPSEQ)
Source: Plant J. 2013 Oct 10;76(4):718–27. doi: 10.1111/tpj.12319 (PMC4298792; doi:10.1111/tpj.12319)
Supplement: Supplementary file 7 — Methods S1. Experimental procedures for Appendix S1. [file tpj0076-0718-sd7.docx]

**Mascher *et al* Supporting Methods S1:**

**Identification of orthologous *Brachypodium* genes**

Protein sequences of barley high confidence genes were downloaded from MIPS Plant DB ([Nussbaumer et al. 2013](#_ENREF_6)) and aligned by reciprocal BLASTP against the protein sequences of Brachypodium gene models (version 1.0) ([International Brachypodium Initiative 2010](#_ENREF_3)). The locations of best bidirectional blast hits were visualized with R.

**Mapping of Vrs1**

Phenotypes for Vrs1 (six-rowed spike) in the OWB population were obtained from GrainGenes ([Carollo et al. 2005](#_ENREF_1)). The OWB population was divided into subgroups of individuals showing either the dominant (two-rowed) or the recessive (six-rowed) phenotype. The allele frequency of the dominant and recessive alleles was computed at each individual GBS marker ([Poland et al. 2012](#_ENREF_7)), averaged in 1 cM bins and visualized along the genetic length in R. The sequence of the Vrs1 gene ([Komatsuda et al. 2007](#_ENREF_4)) was retrieved from NCBI (acc. no. AB259782.1).

**Supporting References**

Carollo V, Matthews DE, Lazo GR, Blake TK, Hummel DD, Lui N, Hane DL, Anderson OD. 2005. GrainGenes 2.0. an improved resource for the small-grains community. *Plant physiology* **139**(2): 643-651.

IBSC. 2012. A physical, genetic and functional sequence assembly of the barley genome. *Nature* **491**(7426): 711-716..

Komatsuda T, Pourkheirandish M, He C, Azhaguvel P, Kanamori H, Perovic D, Stein N, Graner A, Wicker T, Tagiri A et al. 2007. Six-rowed barley originated from a mutation in a homeodomain-leucine zipper I-class homeobox gene. *Proceedings of the National Academy of Sciences of the United States of America* **104**(4): 1424-1429.

Nussbaumer T, Martis MM, Roessner SK, Pfeifer M, Bader KC, Sharma S, Gundlach H, Spannagl M. 2013. MIPS PlantsDB: a database framework for comparative plant genome research. *Nucleic acids research* **41**(Database issue): D1144-1151.

Poland JA, Brown PJ, Sorrells ME, Jannink JL. 2012. Development of high-density genetic maps for barley and wheat using a novel two-enzyme genotyping-by-sequencing approach. *PLoS One* **7**(2): e32253.
